# On Pyæmia

**Published:** 1860-06

**Authors:** H. P. Dewees


// NEW YORK MONTHLY REVIEW

AND

BUFFALO MEDICAL JOURNAL.

VOL. 15. JUNE, 1860. NO. 13.

### ORIGINAL COMMUNICATIONS.

---

*On Pyæmia.* By H. P. Dewees, M.D. (Read before the N. Y. Medico-Chirurgical College, April 12, 1860.)

Gentlemen—At the last meeting of the College, I had intended to open the subject proposed for the discussion of that evening, Pyæmia, but owing to the lateness of the hour, and to the impossibility of exposing my views on a subject of such extensive physiological and pathologic relations, I asked, and received, permission to defer the discussion until this evening, when I should read before you a paper that would define these views, and better admit of subsequent consideration.

The subject of Pyæmia is so extensive in its relations, that its entirety cannot be encompassed in the limits necessarily attached to this paper. The attention of the College, therefore, will only be called to those most prominent pathologic attendants which at different times have been enunciated by me during the last fifteen years. But, before entering upon the discussion of the morbid phenomena, it will perhaps be more satisfactory to state some of the histological and normally mature relations upon which these views are based. And I would desire to be understood, that I do not here mean by Physiology, the study of the laws of life, as commonly defined; but the study of the dynamical relations of organic structures. Thus defined, physiology becomes correlative to histology and minute anatomy. Nor, by

Pathology, do I wish to be understood the study of *dead* structures alone, but rather the study of *dying* structures, since any aberrance from the normal standard of health is a true pathologic progression. It is only by this knowledge of "progressive pathology" that, to the "ars medendi," can be insured a result approximating to certainty. The intercurrent history of any disease is not inscribed by the finger of death. It is to be studied in the early alphabet of commencing disturbance, typed in the numberless examinations of those dying from other causes, but in whom an accompanying disease could be traced from its beginning to its end. It is thus only, by combination from its Alpha to its Omega, that pathology can become the language of disease.

The chyle and lymph are to be regarded as the primary condition and source of the blood. Their elaboration is intrusted to certain glandular actions. The lymph-ducts everywhere accompany the blood-vessels, and pour part of their contents, at least, into the current of the blood, in which the cytoid lymph-corpuscle now becomes the white corpuscle of the blood. During the regeneration of the tissues, as well as in pure lymph exudations, an endogenetic action ensues, by which the corpuscles (now known as the cytoid corpuscles) are developed. These cytoid corpuscles are in every way identical with the white corpuscles of the blood. In their development, they are connected with the organic transformations of the plasma into tissue, and probably are the resultants of the primary regenerative action, by which the albumen of the afforded pabulum is converted into the various structures, by contact with the relative organic substance, in accordance with the law for the reproduction of organized similitudes.

The white corpuscles of the blood are subject to variation, not only as to their constituent composition, but also as to their number in proportion to the red, their density, and their contents. They contain less iron, but more fat, and have distinct nuclei; whilst no true nucleus exists in the red corpuscle. Their proportion to the red seems to vary according to the rapidity of structural demand. In the young, the proportion of the white to the red corpuscle is a third more than in the adult; whilst during menstruation and pregnancy, in which periods an almost similar corpuscular amount exists, the same proportion, when compared to the non-menstruating state, attends.

It would appear, therefore, from these and other facts, that in the normal condition, the white corpuscles are the indicators of the nutrient activity in the lymph itself, as they do not of themselves enter into structural repetitions, but are the products remaining after primary assimilation. They thus indicate by their proportion, diminishing or increasing beyond the normal amount in the blood, one of two conditions. Namely, the supply of food being the same, that the clear nutrient lymph has been consumed by the assimilative demand, with a less amount developed into the cytoid or white corpuscle; or, that the lymph has been supplied too profusely for actions of repair, and has been unassimilated and undergone corpuscular change, their excess being proportioned to the quantity not used. This view is corroborated by the examination of the blood in health, and in the abnormal condition called leucocythemia, in which the white corpuscles are in proportional excess. It is now to be remembered, that the products of the food, the chyle and lymph, are not immediately changed into red blood. Like that poured into the right heart by the thoracic duct, the lymph-vessels pour their contents into the blood-vessels in the various portions of the body, and afterwards the aggregated amount is subjected to the action of the lungs, which in their turn must be nourished, and submit their unused portions to the oxygenating process of the air-cells.

*Development of the Cytoid Corpuscle into the White Corpuscle.*—The white corpuscle is then to be considered as the cytoid lymph-corpuscle, in a state of organic progression. When subjected to the action of the lungs, oxygen is absorbed, and its albumen having received its increment of that element, it is converted into soluble fibrin, whilst the watery contents are displaced, to pass off as vapor from the surfaces of expiration, or remain to hold the salts of the liquor sanguinis in solution. The culminative transition into the red corpuscle now ensues, by the elimination of the nuclear contents of the white corpuscle at once, and their endogenetic development; or by their progressive perfectioning, when submitted to the other organs in which the hæmagenic functions reside.

The red corpuscles, in this manner, owe their origin, in part at least, to the progressive morphoses of the white corpuscles, and hence it may be that the latter are the parents of the former. It is now that the liquor sanguinis is prepared to sustain the nutritive acts, after the active operations of the pure lymph have subsided. Hence we see how every portion of the nutritive supply is converted, and the demand for necessary renewal is made. The fibrillation of the fibrin, held in solution, is a vital act. It is necessary for the formation of the tissues, and when the supply is inadequate, or imperfect, changes of texture must necessarily ensue.

*Transitive Changes of the Liquor Sanguinis.*—The nutritive changes from the liquor sanguinis are effected in the transitive vessels, between the arterial and venous circulation—the capillaries. The now perfected fibrin is evolved in its soluble state, according to the demand for the formative power of the various tissues, whilst the products of dis-assimilation are emptied into the venous radicals, to undergo the depurating processes of the excretory actions of the special organs. If this depurating process is interfered with by the derangement of any of these organs, then the extractive materials which should have been eliminated collect in the blood. Hence, the normality of the organic interchanges is disturbed, and the reductive changes are forced on other organs yet sound, but which may be disordered by this over-tasking of the functions, and by the poisoning or reactive manifestations of the central or terminal portions of the nervous system.

*The Red Corpuscle a Matured Product.*—The red blood-corpuscle is, then, not to be viewed as directly concerned in the regeneration of tissues. They are the matured products of the nutritive act itself. Their chief function lies in their power of absorbing and holding in solution oxygen, and giving off carbonic acid and other gases, generated during the interchanges of nutrition, whereby the heat of the body is, in part, maintained; and this takes place not in the lungs alone, but wherever nutritive reactions ensue.

The liquor in which the red globules are suspended corresponds chemically with the lymph, there being rather less fibrin in the latter, as might be anticipated from the statements just offered, whilst the albumen is richer in the former. Lymph, then, is not to be regarded as derived from the blood itself, viz., the red and white corpuscles and the liquor sanguinis. But the blood is derived from the chyle and lymph. From these, in the human embryo, the blood in the second month is developed. Whatever influence the vessels themselves possess in the elaboration of the blood is not yet satisfactorily explained, although direct absorption by the veins is acknowledged, and may account for the increased quantity of albumen in the liquor sanguinis over that of the lymph.

*Degenerative Cytoid Conditions.*—It is to this disposition of lymph exudations to cytoid development that I would now call attention. It is the key to the comprehension of the formation of pus, and its morbific dissemination, called Pyæmia. As nutrition is compounded of assimilation and disassimilation, so is pus a resultant of degenerated cytoid corpuscles, and of the dissolution of the structures affected. If the condition, known as inflammation, should arise, the amount of local assimilable lymph may be increased at first, but is afterwards diminished. The organic nerves are excited, the capillary vessels become lessened in calibre, fibrin is locally generated, the blood is hurried more rapidly through the part, whilst structural reactions are more or less deranged. Hence, in the very onset of the inflammatory process, disorder of local nutrition is established. In other words, normality—the highest state of vital action—is impaired. The plasma is prevented from being organized into true tissue—the cytoid corpuscle is more or less degraded, and tends, by retro-morphosis, to degenerate into pus, an effete corpuscle, no longer subservient to the uses of the economy, since its arrest of development (from the original cytoid condition) into the white and subsequent red corpuscle is effected. Unless the constitution is vitiated, or the local cause has been greatly depressent, the exuded lymph resists a rapid degeneration into pus, as seen in fresh wounds, wherein this change sometimes is not effected for many hours. The transitional grades may readily be perceived by even the unarmed eye. This persistence of vitality in lymph is taken advantage of in endeavoring to heal a wound by what is termed "first intention." But if the individual be impoverished by disease, or his vitality be lowered, this persistence in the material for repair is lost, the organic cells become more or less vitiated or degraded, the nervous centres, or their terminations, are either irritated or depressed, exterior influences are unrestrained, and pus is early generated; or a sanious ichor proclaims the beginning of local chemical change. In the first condition cited, fibrillation is perfect; whilst in the second, its rapid disengagement (as of the other structural portions) into carbureted and sulphureted hydrogen, announces that chemical change is stronger than vital resistance. These are cases where pus has an exterior exit—not, as in pyogenic formations, inclosed in deep cellular or parenchymatous organs, where natural opening or surgical equalization, by the knife, cannot be attained.

*Action of Nature in Pus Formations.*—The action of nature is different in these cases. When art cannot reach the part, the pus remains localized as an abscess in the structure, or it is deposited in the cellular spaces; and its removal is to be effected, either by its being carried by chance into some patulous-venous orifice, with future impedimentary risk, or, by a series of auxiliary efforts of the capillaries and absorbents. The fluid portions are taken up; the granular portions, if disintegrated, enter the patent capillary vessels, (the dilating influence of the emitted gases, perhaps, aiding,) and their transmutations into excrementitious matter with excretory disengagement, may or may not ensue.

It is worthy of remark, that the retained pus-corpuscles of abscesses are smaller than those from open wounds. Pus contains not only an albuminous material, but its granules are largely compounded of fat. Hence the emaciation which follows large suppurations.

If the disintegration of pus into its molecular basis, is not perfected in its transition to and through the excretory organs; in other words, if the lungs, liver, kidney, and other excretory organs and surfaces cannot resolve them into excrementitious, gaseous, or fluid excretions, the portions so incarcerated may become the foci of disorder of local nutrition, and thus superinduce pyæmic changes. It is thus that structural changes ensue in an organ, distal to the originating point of suppuration. Generally, the induced derangements are more of function than from absolute decadence of structure. Yet this sometimes does ensue, not in one organ or locality, but in several; and thus pyæmia is established. But it is the pyæmia of the structures, and not the pyæmia of the blood, in which the white corpuscles are everywhere degraded by a *toxic* element; and the red, long unfit for their normal uses in the economy, are in a condition approaching dissolution and decay.

It is to be remembered that the prominent outlines of pyæmic formations are only delineated, and that I am not describing the various processes of inflammation. This subject will be more fully entered on by our Fellow, Prof. Peaslee, in his following paper.

*Corpuscular Pus never Absorbed.*—Pyæmia, as now generally understood, is owing to the retention, dissemination, or generation of purulent or putrescent matter in the blood. True pus (i. e., in its corpuscular state) is never absorbed, as has been often stated. Artificial pyæmia may be caused by the accidental or intentional introduction of extraneous substances, with their subsequent arrest in the smaller vessels or capillaries. If phlebitis, or capillary inflammation is established, the coagulation of the blood in the injured vein may ensue, as an apparent means or attempt at arrest of further morbific propagation. The clot may soften, shrink, and dissolve in part into a pus-like fluid, leaving, after the absorption of the watery portions, a debris of mixed albuminous material, sometimes resembling tubercle. Or infarction of the capillaries may ensue, (sometimes designated "apoplectic spots,") with local and surrounding textural degeneration into pus, the severity of the symptoms depending chiefly on the quality of the disorganizing mass, the constitutional conditions, and the organic value of the part itself. But this is a mere artificial pyæmia—innocent in its general conditions. It is not a *septicæmia*, in which the element is toxic and infectious. One partakes more of the nature of an infiltration; whilst the other is a true purulent infection. For we many times have phlebitis attended with obstruction without pus infection.

*Fibrinous Concretions.*—Consolidated fibrin may break off from the valves of the heart, or from other parts, and be arrested in the pulmonic or systemic vessels, according to its location; and although it may soften and be dissolved into a purulent fluid, still the symptoms and the danger are more of obstruction than of contamination, and more of situation than of pathologic propagation. If located in the small vessels of the heart itself, giving rise to abscess, with discharge of pus into its chambers, or into the large vessels, coagulation may ensue, and life be suddenly cut short. Here the pus is impedimentary—not poisonous; or, the concretion may be carried into the lungs, giving rise to lobular congestion, or capillary apoplexy, with subsequent pus-generation; or, it may be carried into the brain, producing derangements of intellect, of sensation, or of motion. In all these cases, though pus may be produced, and the organs more or less deranged, yet the symptoms and the danger to life are more from local injury than from debasement of the vital force from purulent infection. But if the vitality of the blood has been lowered conjointly with that of the nervous system, then the local pathologic conditions are altered, sloughing or gangrenous dissolution may ensue, and thus a pyæmia be established, both local and general; and many of these cases pass for rheumatism, pleurisy, pneumonia, and other disorders, having "*itis*" as their termination, and have been consequently treated by depletory remedies, when the treatment should have been sustentative.

*Preference of Pyæmic Locations.*—The lungs are more prone, in pyæmia, to be early affected, as the purulent or septic materies is thrown into the right side of the heart, and thence into the pulmonic capillaries. Their physiological position as eliminators of morbid blood products, and the vital affinity that attracts to them the white corpuscles, especially for oxygenating purposes, offer the best reasons for the frequency of this pathologic impairment. If the morbific material has been forced on to the systemic side of the heart, the brain, liver, spleen, kidneys, &c., may become the seat of the affection. Another source, however, may arise, especially in injuries below the chest, from the introduction or arrest of the pus or septic animal matter, in the portal system, by the returning sub-venous currents; and this may be perfectly independent of any phlebitis of the immediate portal circulation, or of inflammation of the hepatic artery.

*Pyæmic Pervasion.*—A third form of pyæmia may arise, wherein every portion of the body becomes affected—lungs, heart, liver, spleen, serous membranes, muscular structure and surface. And this grave condition ensues not so much from local purulent absorption *in se*, as by the degradation of every tissue, from degenerative elective actions of local nutrition, and from impurity of the plasma afforded. Unless we have been able to trace the early history of the patient's condition, an error is not unapt to be made in the diagnosis. Since our attention is more directly called to the patent symptoms of inflammation of the serous membranes, such as pleurisy, peritonitis, &c.; or when in parenchymatous organs, to the lungs, liver and spleen, from the exudative reactions offering in these parts. But nature here points out a most valuable lesson as regards treatment, but which our fathers neglected, as well as some of ourselves continue to do. The exudation, or so-called inflammatory product, *although lower than the normal nutritive plasma*, is yet higher than pus, and into this it would degenerate by any lowering plan of treatment. Both physiology and pathology dictate the contrary. The patient must be sustained by food, and alcohol especially, which has the power, as has been before stated by me, of arresting the too rapid decomposition of albuminous compounds.

In many of these cases there is no pericarditis from bursting of an abscess in the outer wall of the heart; there is no peritonitis from matter irrupting into the abdomen; or from a suppurating liver. There is no localization, no arrest with disintegration, or restrictive exudation; every organ is poisoned, and death results from the pervasion of molecular disorder of the whole fabric. And what does morbid anatomy show in autopsy? Sometimes absolutely nothing to the naked eye, save a few ecchymotic spots here and there, but no great depots of putrid pus; no ulcers with circumscribed borders, to show the effort of nature towards reparation. There is no witness standing forth from the dead organs; yet, in every fibre and in every molecule, there lurks the hidden attestor, proclaiming that the pathology must be studied in the *dying* as well as in the dead structures.

*Superficial Pyæmic Elimination.*—But the internal organs do not always bear the brunt of the disorder. The skin may either become the eliminating depot, the channel for blood depuration; or it may be the locale of the toxic introduction. This may happen in several ways: 1st. The natural eliminative capacity may reside in the surface. 2d. The septic introduction, or delay, may be in the integumentary capillaries, or in the superficial lymphatics. 3d. The peripheral nervous system may be paralyzed, or poisoned.

The prognosis in these cases is more favorable, in many respects, than in internal pyæmia; since the bursting of an abscess externally, or superficial ulcerations, are comparatively harmless as to contents and local value, whilst the treatment can be better applied and watched as to effect.

*Pyæmia of the Joints.*—There are other surfaces in which pyæmic exhibition or infection ensues; namely, the joints. As mentioned by me in my article on "Rheumatism of the Epithelial and Non-epithelial Tissues," the joints are more prone to become affected when the kidney is diseased, and especially after exanthematous disorders.

The joints are also liable to suppurative destruction, when the internal structure of a bone is injured or diseased. Hence, after amputations and fractures, especially compound fractures, with ends of bone more or less exposed, the disposition to pyæmia is most frequent. Statistics show that nearly one-half of the unfavorable results after amputation arise from pyæmia. The tissue operations are comparatively exempt from this complication, if are excepted those of perineal section of stricture, of lithotomy, and of the rectum. In post-puerperal states, the sterno-clavicular joint not unfrequently becomes affected, as stated by Dr. Todd, from pyæmic disturbance, with abscesses extending into the surrounding muscular structures. *The symptoms and cause are generally mistaken for those of rheumatism.*

Amongst what might be termed the natural causes of pyæmia, the most frequent arise from abscess in the leg, or purulent septic matter taken up from the uterus and the circumjacent pelvic organs. But bone seems a most favoring source for the transmission (if not for the absorption) of the purulent poison. The cancellous structures and haversian canals offer a ready conduction, whilst the stationary calibre of the vessels, from their adherence to the sides of their conducting canals, affords an additional source of entrance into the circulation. It is very certain that after injury, or other textural disturbance of the bones of the head especially, pyæmia is by no means an unfrequent complication. And, as before mentioned, the seat of the purulent retention is generally in the lungs and its serous coverings; whilst death may suddenly ensue from the coagulation of the blood, rapidly produced by some forms of pus, even in the interior of the heart itself. The shock to the nervous centres, by the blow or other causes, however, comes in for a participation in the pyæmic formations. In these latter cases, the depositions result more directly from the disturbance of the nutrition of the tissues themselves, than from *thrombosis* or embolic arrests, ushered in by coagulation or capillary apoplexies. These cases, whether from absorption of the toxic elements of pus, or from depraved nervous changes over nutrition, are most resistant to treatment; since the fibrin is either not evolved, or it is of a soft flaky kind, indisposed to fibrillate, and readily breaks down into a pus-like fluid. The disorder, when so located, is frequently termed lobular pneumonia. In many cases of pyæmic localization in the lung-tissue, and its serous covering, as well as when in the joints or their neighborhood, I have known it diagnosticated as rheumatism, the *pain*, and not the *process*, being diagnostically considered; and such, I understand, was the belief in the case of one of our late eminent physicians.

*Influence of Suppurative Surfaces in the Debilitated.*—During low states of the constitution, any suppurative act may induce the pyæmic condition. It has followed gonorrhœa in the already debilitated, and especially if accompanied by perineal abscess. Though the vessels are anatomically more patulous in this region than in many others, yet the septic influences depend more on the particular diathesis than on the mere admission of pus itself into the larger vessels, or by its capillary reduction. Typhoid fever is also apt to induce pyæmia, by the absorption of the half-putrid contents of the bowels from glandular ulcerations and sloughing, as well as from the inability of these glands to depurate the blood from its excrementitious impurity. Sometimes the mucous membranes throw off vast quantities of puriform matter, and hence arose, in great measure, the term "secretion of pus." But pus is never secreted—it is an effete material, and as such it is excreted, as no longer serving vital uses. For pus is a product of change—it is not a process—it is the de-vitalized result of blasted organization, and hence it is neither secreted, nor has it the power of organic re-formation. In those cases of erysipelas attended by suppuration, but which may suddenly, in the progress of apparent cure, eventuate into pyæmic symptoms, the constitutional damage (in many individuals) has been antecedent to the erysipelatous irruption, from more or less chronic disorder of the kidney especially; the lowering of the nutritive assimilations being attributable more to the uræmic condition, than to any special toxic infection from the absorbed pus.

In considering this subject, the space allotted me has been encroached upon by the histological, physiological, and pathologic recital of the normal and abnormal blood conditions, with some of the many existing structural changes. The remaining space will be occupied by a more condensed exposure of my views on the *rationale* of treatment.

*Treatment.*—This should be decidedly sustentative. But here comes the difficulty, to know how much animalized food can be safely borne, and how much hydro-carbon can be advantageously administered in the shape of alcohol. I say *safely* borne, since it must be remembered that the blood is already overcharged with the animal products of decaying pus and rapidly wasting tissues. Assimilation and assimilative power are so brought to their lowest conditions, that the lymph and cytoid corpuscles are robbed of their normal capacity, and have impressed on them a predisposition to assume lower organic forms, down to pus itself. It is thus that pus begets pus.

The organic balances, then, are to be accurately studied, to determine the exactitude of treatment. If the eliminatory organs are equally impaired, or are equally overworked, alimentation and alcoholization must be proportioned, so as to keep the remaining constitutional power at the *effort guage* it is capable of working under. Above or below this standard is injurious, or tending to fatal result. For if above, the nervous system is forced beyond its legitimate condition for recuperation; if below, besides the increasing blood depravity, it is subject to molecular degradation in its own structure. Generally, this proportionate equality in the standard of the eliminatory organs does not attend. The kidney may be defective, whilst the lungs are comparatively sound, and making great effort to throw off, by ammonia, carbonic acid, and vapor, the additional amount imposed on them for elimination. Or the liver may have been submerged in its functions, and with a defective kidney, add to the vast constitutional poisoning by the imperfect excretion of bile so essential to primary nutrition, and by the non-excretion of the products from the blood, now pus-poisoned.

Thus, there is no exact constitutional level; assimilation is at a low ebb; reproduction is fast yielding to destructive resolution; and chemical changes threaten the whole system. What can be done to cause this havoc to stand still, or that will drive death beyond the precincts of life? Will alcohol, a nervo-stimulant, but a hydro-carbon, do it? Will it, in a blood already surcharged with the destructive products of disassimilation, and the emunctory organs paralyzed in their function, will it form the remedy to sustain life, or will it fail to strengthen and repair? The lungs, laboring like some Vulcan at the bellows, can they escape the immutable law that assigns a doom to every organ overtaxed in its functions? Can they live through the deadly corruption that attraction fastens on their textures?

These are questions that must arise in the study of the treatment; and, although the broad basis of sustentation must be stood upon, still it becomes the question, what other remedy will keep the vital powers in their normal action, without adding to the load of the re tained elements now overwhelming the system ? Is alcohol the only platform upon which the foot of success can rest ? Or will other remedies, having no oppressive re-formations, and whose bulks do not inconvenience the stomach, whilst their influence is special and tonic, but not tumultuous, will they in practical result, as well as in theory, sustain the powers of life better, or even as well, as alcoholized remedies ?

It is to be remembered that I am speaking of those cases in which the great eliminatory organs are either diseased or paralyzed in their functions. There is no doubt, where these organs can sustain the elimination of the plusages of carbon, &c., that alcohol forms a most trusty remedy. It is not so much by the sustentation of the nervous system, as by its affording free carbon for oxygenation, in place of that combined in the tissues, especially in the fat so essential to the brain and nerve-formation; and also by its inherent property of retarding the too early retro-morphosis of albumen, which, as stated by Prof. Peaslee, is the *pabulum* of the tissues. But I have cited cases where the impediment to functional excretory action is great; where, besides carbonic acid, the lungs exhale putrid nitrogenized matter, held soluble in their vapor, and tainting the breath with its descriptive fœtor. Thus the stone of Sisyphus,

"Ingens et non exsuperabile saxum,"

is ever rolling back on the weakening arm of life. Have we in bark, in its alkaloids of quinia, chinoidine, &c., in arsenic, in corrosive sublimate, and the like, have we properties of sustentation as well as of molecular septic retardation ? It is only by such investigation and faithful observations that medicine can make its approach to a scientific basis. For a man may be as thoroughly drowned in his own fluids—the great centres of life, circulation, innervation and respiration, may be as surely submerged and extinguished by induced internal conditions, as though the individual were sunk full fifty fathoms deep.

*Fact vs. Theory.*—But these theories, so expressed, are more specious than feasible. It is to be remembered that the blood is not burdened with the materials of assimilation, but with the effete and putrescent products of *dis*-assimilation. The waste is greater than the repair—and that waste is not an organic debris, but a poison. Then alcohol, in *regulated* quantities, is not only a food, but it becomes an arrester of dissolution and decay. It is to *alimentation* that our attention and care must be turned. For more die from the overcrowding of nitrogenized food than of carbonized remedies. The beef-tea, and not the brandy, is to be watched. Oils, such as the cod-liver, are chemically and assimilably demanded; they supply the waste, whilst the alcohol, by its stimulus, sustains the nervous system, till the eliminative actions are consummated.

*Alcoholismus.*—It is true that the drunkard's acne-covered face, his bloated and ulcered limbs, his slavering mouth, and drowsy brain, seem to point the finger of distrust towards alcohol as a remedy in the pus-poisoned. But it must be remembered how, from neglect of cleanliness, of hygiene, of sleep in the mid-hours of the night, when stupor wastes and makes him wander, how this man of many errors falls under the ban of nature's broken laws—a bankrupt in all health. He is the poisoned witness of every debauchery, and of the *abuse* of alcohol; and acne, and ulcers, and atrophied peripheral nerves, both organic and sensory, tell more the tale of many vices than of one alone!

We have now considered some of the chief forms of pyæmia. One induced by pus localization—comparatively benign in its influence, although attended by disorder of local nutrition; the other, a true pyæmia or septicæmia, from the breaking down of exudation, with an additional internal toxic element from decay or putrefaction.

*Cadaveric Pyæmia.*—But there remains a third form from external introduction, as from dissecting and other wounds possessing a multiplying septic element. It is the pyæmia of inoculation, specific in its character. Like inoculation from variola, equinia, and syphilis, it produces certain changes in the nutritive fluids, or cells; but it is not subject to their type-life conditions of regularity, of duration, and manifestation. The retro-morphic changes generally are more persistent, the eventuations more hazardous, and the recovery therefore more tedious, and less frequent.

I will not further occupy the attention of the College; and in conclusion, will merely mention that I have intentionally refrained from dilating on the pyæmic complications of puerperal fever, as their consideration would call for more time than could be granted at this session. Nor have I given place to the symptomatology of pyæmia, as I did not consider it necessary to the intention of this article, and as the prominent features were known to all, whilst the discussion of those of the occult or mixed character would draw still more on your time and patience, now already overtaxed.

791 Broadway.

